# Osteogenic differentiation of preosteoblasts on a hemostatic gelatin sponge

**DOI:** 10.1038/srep32884

**Published:** 2016-09-12

**Authors:** Zong-Keng Kuo, Po-Liang Lai, Elsie Khai-Woon Toh, Cheng-Hsi Weng, Hsiang-Wen Tseng, Pei-Zen Chang, Chih-Chen Chen, Chao-Min Cheng

**Affiliations:** 1 Institute of Nanoengineering and Microsystems, National Tsing Hua University, Hsinchu City 30013, Taiwan; 2 Pharmacodynamics Technology Department, Center of Excellence for Drug Development, Biomedical Technology and Device Research Labs, Industrial Technology Research Institute, Hsinchu City 30011, Taiwan; 3 Department of Orthopedic Surgery, Bone and Joint Research Center, Chang Gung Memorial Hospital, College of Medicine, Chang Gung University, Taoyuan 33305, Taiwan; 4 Institute of Applied Mechanics, National Taiwan University, Taipei City 10617, Taiwan; 5 Institute of Biomedical Engineering, National Tsing Hua University, Hsinchu City 30013, Taiwan

## Abstract

Bone tissue engineering provides many advantages for repairing skeletal defects. Although many different kinds of biomaterials have been used for bone tissue engineering, safety issues must be considered when using them in a clinical setting. In this study, we examined the effects of using a common clinical item, a hemostatic gelatin sponge, as a scaffold for bone tissue engineering. The use of such a clinically acceptable item may hasten the translational lag from laboratory to clinical studies. We performed both degradation and biocompatibility studies on the hemostatic gelatin sponge, and cultured preosteoblasts within the sponge scaffold to demonstrate its osteogenic differentiation potential. In degradation assays, the gelatin sponge demonstrated good stability after being immersed in PBS for 8 weeks (losing only about 10% of its net weight and about 54% decrease of mechanical strength), but pepsin and collagenases readily biodegraded it. The gelatin sponge demonstrated good biocompatibility to preosteoblasts as demonstrated by MTT assay, confocal microscopy, and scanning electron microscopy. Furthermore, osteogenic differentiation and the migration of preosteoblasts, elevated alkaline phosphatase activity, and *in vitro* mineralization were observed within the scaffold structure. Each of these results indicates that the hemostatic gelatin sponge is a suitable scaffold for bone tissue engineering.

To hasten the translational lag from laboratory to clinical studies in bone tissue engineering, we used a hemostatic gelatin sponge, a longstanding US Food and Drug Administration (USFDA)-approved material, as a scaffold to repair bone defects. Skeletal defects, which can be caused by irradiation, trauma, nonunion, disease (e.g., osteoporosis), and/or tumor resection, require complicated reconstruction efforts using bone grafts^1^,^2^. Bone graft source approaches commonly include autografts, allografts, artificial bones, and more. Autografts, the current gold standard for bone graft procedures, are used to enhance bone-healing, spinal fusion, and fracture repair. However, autografts require a secondary operation to remove material from the donor site, which increases postoperative pain and impacts surgical success. Allografts, often cadaver tissue, do not require a secondary operation, but there is a limited supply of material and a minimal but genuine risk of disease transmission^3^. Engineered artificial bones are a very viable alternative because they are durable, biocompatible, osteoconductive, and osteoinductive^1^,^4^,^5^.

Bone tissue engineering has been studied for many years. Many factors, including cell source, signaling molecules, scaffold biomaterial characteristics, and culture conditions, have been widely investigated with the aim of successful bone tissue engineering^2^,^4^,^6^,^7^. Preosteoblasts, precursor cells to osteoblasts, are important for bone formation; they regulate mineralization and the expression of functional proteins such as alkaline phosphatase (ALP) and osteocalcin, which are critical components of collagen production^8^,^9^. In addition, osteoblasts differentiate into mature osteocytes, which generate syncytial networks and support bone structure and metabolism. Preosteoblasts, osteoblasts, and other sources of osteoprogenitors have been widely used for bone tissue engineering^10^,^11^,^12^. Moreover, various biomaterial scaffolds have been employed to provide structural support and provide an environment for osteogenic differentiation; these scaffolds can even have signaling molecules incorporated into them to promote repair and regeneration^2^,^13^. Synthetic polymers, e.g., biodegradable polyesters poly (lactic-co-glycolic acid) (PLGA) and polycaprolactone (PCL), have been widely applied and investigated as scaffolds for bone tissue engineering^14^,^15^,^16^. Naturally derived materials, including collagen^17^ and gelatin^18^, have also been used; these materials have demonstrated suitable biocompatibility and are widely applied in tissue engineering. Although many different kinds of biomaterials have been applied for bone tissue engineering in laboratory studies, biodegradation and biocompatibility must be considered when using these biomaterials clinically^19^.

In order to decrease the safety concerns and ameliorate the translational gap between laboratory studies and clinical applications, several biomaterials and related products widely used in clinical applications can be investigated for their potential as bone tissue engineering scaffolds. Here, we examine the use of hemostatic gelatin sponges in just such a role. Hemostatic gelatin sponges are sterile, water-insoluble, malleable, and absorbable. They are easily obtained, inexpensive, biocompatible, and are not known to induce allergic reactions or other harmful side effects^20^. Hemostatic gelatin sponges have been demonstrated as a suitable *in vitro* model for producing 3-dimensional (3D) human and bovine chondrocyte cultures^21^,^22^,^23^. While some studies have demonstrated the usefulness of hemostatic gelatin sponges as a carrier or an implant for repairing gingival depressions and bone defects^24^,^25^,^26^,^27^, these studies only demonstrated the suitability of gelatin sponge as a carrier or an implant for bone regeneration. For example, Arias-Gallo *et al*. used gelatin sponges as a carrier of fibroblast growth factors^26^ and Skogh *et al*. applied gelatin sponges as an implant for bone regeneration^27^. These studies did not demonstrate their usefulness in regards to cell differentiation. To fully leverage the potential of hemostatic gelatin sponges as a scaffold for tissue engineering, this study has a dual purpose: (1) to examine and report on the physical characteristics of the clinically used hemostatic gelatin sponge, Spongostan; (2) to lay some groundwork regarding the use of this hemostatic gelatin sponge as a scaffold for osteogenic cell differentiation.

## Results

### Biodegradation of the Hemostatic Gelatin Sponge

**Figure 1:**
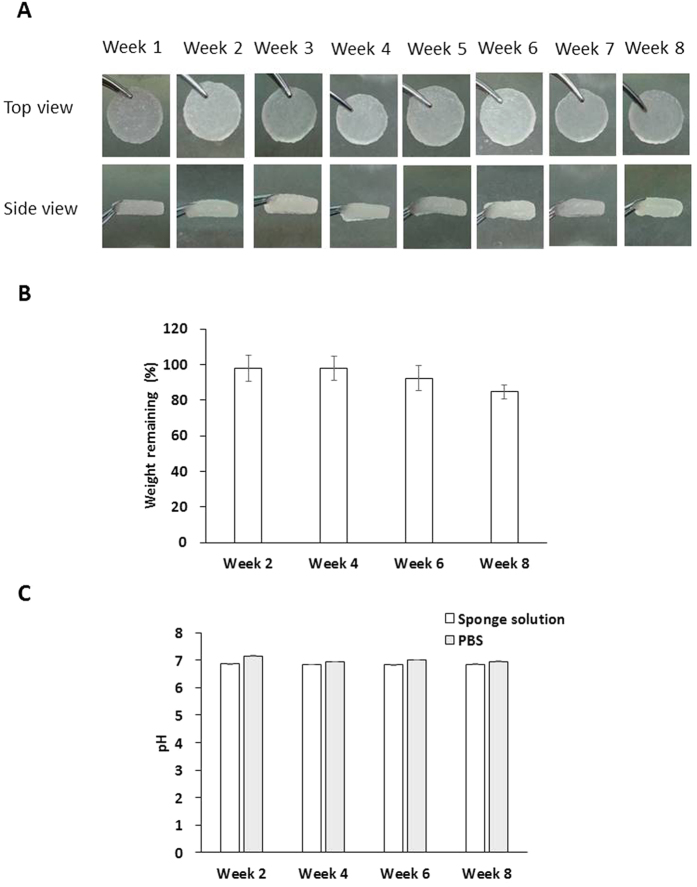
The degradation of the hemostatic gelatin sponge when immersed in PBS. (**A**) Photographs of the sponge at different time points. The hemostatic gelatin sponge maintained its structure for 8 weeks. (**B**) The remaining weight of the sponge after immersion in PBS. The resulting weight at week 8 shows that the sponge maintained 84.9 ± 3.9% of its original weight. (**C**) The pH of the solution around the sponge. The pH was 6.86 ± 0.01 at the 8th week (n = 6).

To examine biodegredation, sponge disks were immersed in phosphate-buffered saline (PBS) and maintained in an incubator at 37 °C for 8 weeks. Hemostatic gelatin sponge condition was observed each week (Fig. 1A). No structural decay was observed, suggesting sponge structure stability under these conditions. The net weight of the sponge and the pH change of PBS were also monitored every two weeks. The remaining weight of the sponge decreased over time (Fig. 1B); however, the sponge weight at week 8 showed that it had maintained 84.9 ± 3.9% of its original weight. The pH changed during this time span from 6.87 ± 0.01 to 6.86 ± 0.01 (Fig. 1C). The pH of PBS without a sponge was 6.96 ± 0.02 at the end of study, a change that may be attributable to dissolution of CO_2_ in PBS (Original pH of PBS is 7.32 ± 0.02). Additionally, we also examined the biodegradation of sponge disks in ddH_2_O. Similar to our observations when using PBS, the sponge disks maintained their structure and final disk weight was 83.7 ± 2.9% of original disk weight after immersion in ddH_2_O for 8 weeks (Supplemental Fig. 1A,B). The pH changed during this time span from 4.41 ± 0.02 to 4.63 ± 0.01 (Supplemental Fig. 1C), which is lower than the pH change observed for fluid media without a sponge (5.13 ±  0.01). As with the use of PBS, the pH change may be attributable to dissolution of CO_2_ in ddH_2_O. However, a higher acidification of ddH_2_O exposed to a sponge was observed when compared to using PBS, which is likely attributable to the higher buffer ability of PBS.

**Figure 2:**
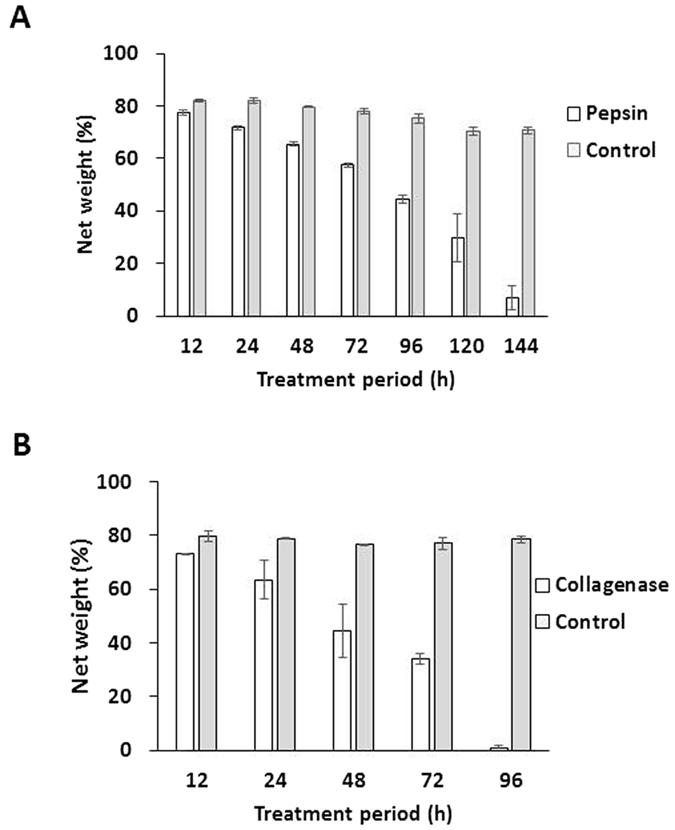
Homeostatic gelatin sponge biodegradation. (**A**) The resulting hemostatic gelatin sponge weight following 10 ng/mL pepsin digestion for indicated time. The remaining weight decreased as digestion time increased. (**B**) The remaining weight of the sponge after 1 μg/mL collagen digestion for indicated time. The hemostatic gelatin sponge was almost digested in this condition after 96 hours (n = 3).

To examine biodegradation further, pepsin and collagenase were used as proteolytic enzymes to mimic the biodegradation of the hemostatic gelatin sponge *in vivo*^28^. Following immersion in pepsin solution at 37 °C, sponges decreased in weight after 144 hours to only 6.8 ± 4.6% of their original weight (Fig. 2A). Collagenase also biodegraded the gelatin sponge. After 96 hours in collagenase solution, sponges weighed only 0.93 ± 0.67% of their original weight (Fig. 2B). These results indicate the biodegradation of the hemostatic gelatin sponge by pepsin and collagenase.

### Compressive Moduli of the Hemostatic Gelatin Sponge

**Figure 3:**
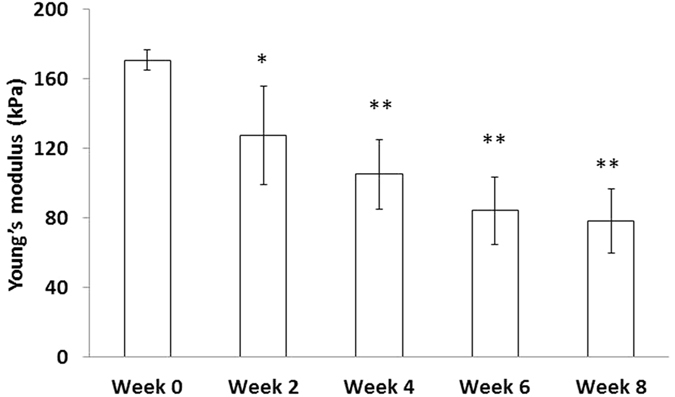
The compressive Young’s modulus of the hemostatic gelatin sponge immersed in PBS for 8 weeks. The Young’s modulus of the hemostatic gelatin sponge decreased as incubation time increased. (n = 6. *P < 0.05; **P < 0.01 compared with week 0).

In addition to monitoring hemostatic gelatin sponge degradation, we conducted compression tests and calculated compressive Young’s modulus to describe mechanical changes of the hemostatic gelatin sponge when immersed in PBS. In the beginning of the compression test, the Young’s modulus of the hemostatic gelatin sponge was 170.81 ± 5.87 kPa (Fig. 3). However, the Young’s modulus of the hemostatic gelatin sponge decreased as the incubation time increased (Fig. 3). The Young’s modulus of the gelatin sponge was 78.39 ± 18.65 kPa at week 8, indicating an approximate 54% decrease of compression strength the gelatin sponge after immersion in PBS for 8 weeks.

### Biocompatibility assay

**Figure 4:**
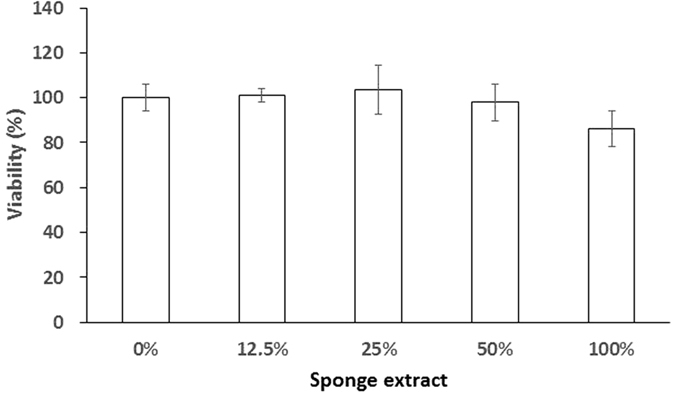
The biocompatibility of the hemostatic gelatin sponge. The viability of preosteoblasts treated with a serial array of extracts from our hemostatic gelatin sponge. MTT assay was conducted to determine viability of preosteoblasts and no obvious cytotoxicity was observed (n = 3).

Generally speaking, high biocompatibility can be considered a reduced interference between material and biological systems, such as cells. To evaluate the biocompatibility of the hemostatic gelatin sponge, we examined *in vitro* cytotoxicity (modified ISO 10993-5) of preosteoblasts using 3- (4,5-cimethylthiazol-2-y)-2,5-diphenyl tetrazolium bromide (MTT) assay. When exposed to serial dilutions of extracts from the hemostatic gelatin sponge, preosteoblasts demonstrated viability higher than 85% for every dilution (Fig. 4). According to ISO 10993-5, viability less than 70% is considered cytotoxic. ISO 10993-5 also states that viability when using a 50% extract should be the same or higher than viability when using a 100% extract. According to both criteria, the hemostatic gelatin sponge demonstrates suitable biocompatibility for preosteoblasts.

### Cell attachment

**Figure 5:**
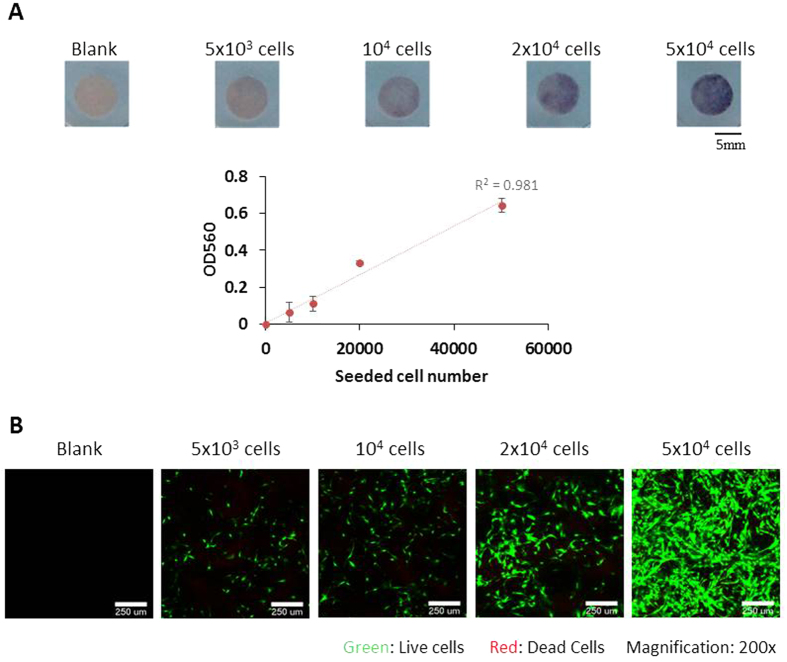
The attachment of preosteoblasts on the hemostatic gelatin sponge. (**A**) Using MTT assay to quantify the attachment of preosteoblasts to the hemostatic gelatin sponge. The color intensity of MTT is proportional to the seeded number of cells (n = 3). (**B**) The attachment of preosteoblasts to the sponge was observed by LIVE/DEAD staining (green: live cells; red: dead cells). The number of live cells increased proportionally to the seeded number of preosteoblasts.

We further investigated the suitability of our hemostatic gelatin sponge for cell attachment. A serial number of preosteoblasts were seeded onto the sponge and cultured for one day. Cell attachment was monitored via MTT assay, confocal microscopy, and scanning electron microscopy (SEM). The MTT assay (Fig. 5A) demonstrates good quantitative linearity, indicating that preosteoblast attachment to the sponge was proportional to the originally seeded cell number.

In addition, the confocal images of the hemostatic gelatin sponge show that the florescent signal of preosteoblasts increased as the seeded cell number increased (Fig. 5B). Live preosteoblasts showed elongated and fractal morphology and few dead cells were observed, indicating that preosteoblasts attached well to the hemostatic gelatin sponge.

### Cell morphology on the hemostatic gelatin sponge

**Figure 6:**
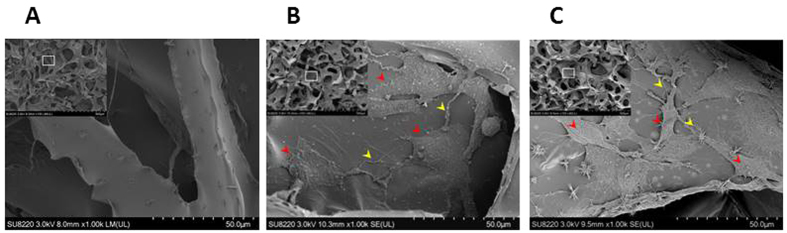
The structure the hemostatic gelatin sponge and cell morphology observed via SEM. (**A**) The structure of the sponge without preosteoblasts. (**B**) The sponge seeded with preosteoblasts using MM. Preosteoblasts demonstrated outgrown morphology, cellular extension and a network between preosteoblasts. (**C**) The sponge seeded with preosteoblasts using OIM. Similar but less spread-out morphology was observed compared with that when using MM. Red arrows indicate preosteoblasts. Yellow arrows indicate cellular extension and a network between preosteoblasts. (MM: maintained culture medium; OIM: osteogenic induction medium).

SEM images showing the structure of the hemostatic gelatin sponge (Fig. 6A) indicate that mean pore diameter was 148 ± 62 μm. In addition, the morphology of preosteoblasts on the sponge was also investigated. SEM images indicate that preosteoblasts spread on the sponge while displaying outgrown morphology (Fig. 6B). In addition, cellular extension and a network between preosteoblasts can be observed. Preosteoblasts in osteogenic induction medium (OIM) demonstrated similar outgrown morphology, cellular extension, and a network between preosteoblasts compared with preosteoblasts in maintained culture medium (MM) (Fig. 6C). However, the morphology of preosteoblasts in MM was more spread out than the morphology of preosteoblasts in OIM. These results indicate that preosteoblasts can attach well to the hemostatic gelatin sponge and the environment of the sponge encourages morphological advancement.

### Osteogenic differentiation of preosteoblasts

**Figure 7:**
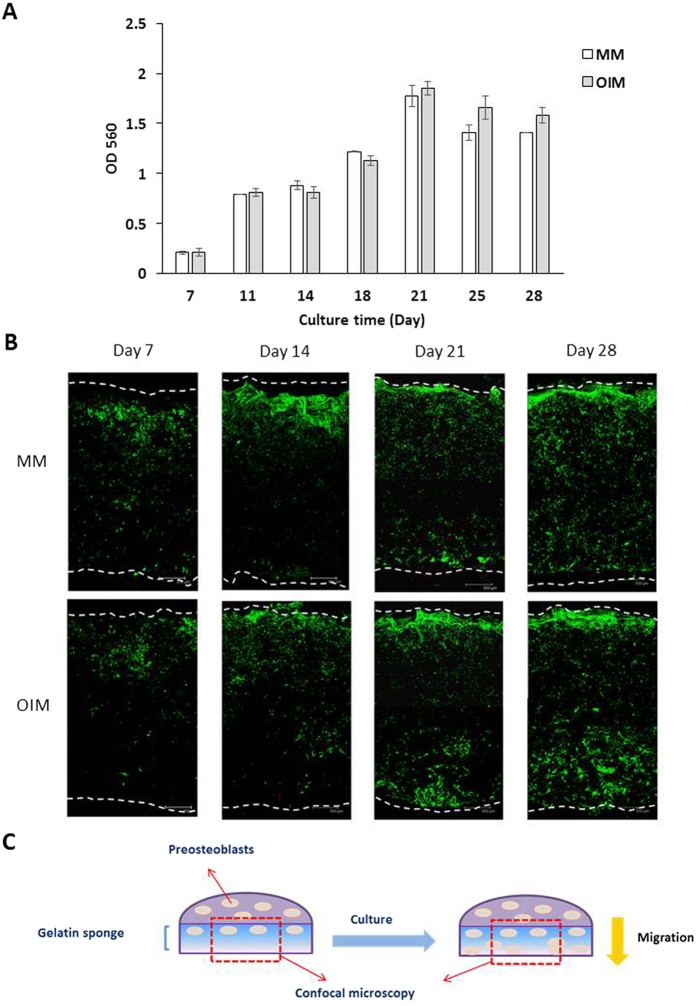
The growth of preosteoblasts on the hemostatic gelatin sponge under osteogenic induction. (**A**) The growth of preosteoblasts was quantified by MTT assay. The growth of preosteoblasts plateaued after 21 days (n = 3). (**B**) Using confocal microscopy to monitor the cross section of the sponge. The downward migration of preosteoblasts from the top to the bottom of the sponge was observed after 21 days. On day 28, more preosteoblasts were observed near the surface of the gelatin sponge than that in the center. The white dotted line indicates the boundary of the sponge. (MM: maintained culture medium; OIM: osteogenic induction medium). (**C**) Schematic illustration of preosteoblastsmigrating within the sponge after culturing for 28 days.

On the heels of encouraging morphological findings, we further investigated osteogenic differentiation of preosteoblasts on the hemostatic gelatin sponge. We seeded preosteoblasts on the gelatin sponge at a density of 10^4^ cells/cm^2^, and we cultured them in OIM and MM while monitoring their growth and osteogenic biomarkers, including ALP activity and calcium deposition, at different time points. The growth of preosteoblasts on the sponge was monitored via MTT assay, which demonstrated that preosteoblasts proliferated as culture period increased, reaching their plateau on day 21 (Fig. 7A). No cell growth differences were discernible between preosteoblasts cultured in OIM or MM.

We also observed preosteoblast distribution (in OIM and MM) along the cross section of the hemostatic gelatin sponge using confocal microscopy (Fig. 7B). Because preosteoblasts were seeded onto the top surface of the sponge, more preosteoblasts were observed near the top surface on day 7 and day 14. Some preosteoblasts were observed inside the sponge, and fewer preosteoblasts were found near the lower surface of the sponge on day 7 and day 14. Interestingly, more preosteoblasts migrated toward the lower part of the sponge as culture duration increased (day 21 and 28). In addition, more preosteoblasts near the lower surface of the sponge were found when cells were cultured in OIM, suggesting that preosteoblast migration can be improved by osteogenic induction. These results indicate migration of preosteoblasts was observed within the sponge (Fig. 7C).

**Figure 8:**
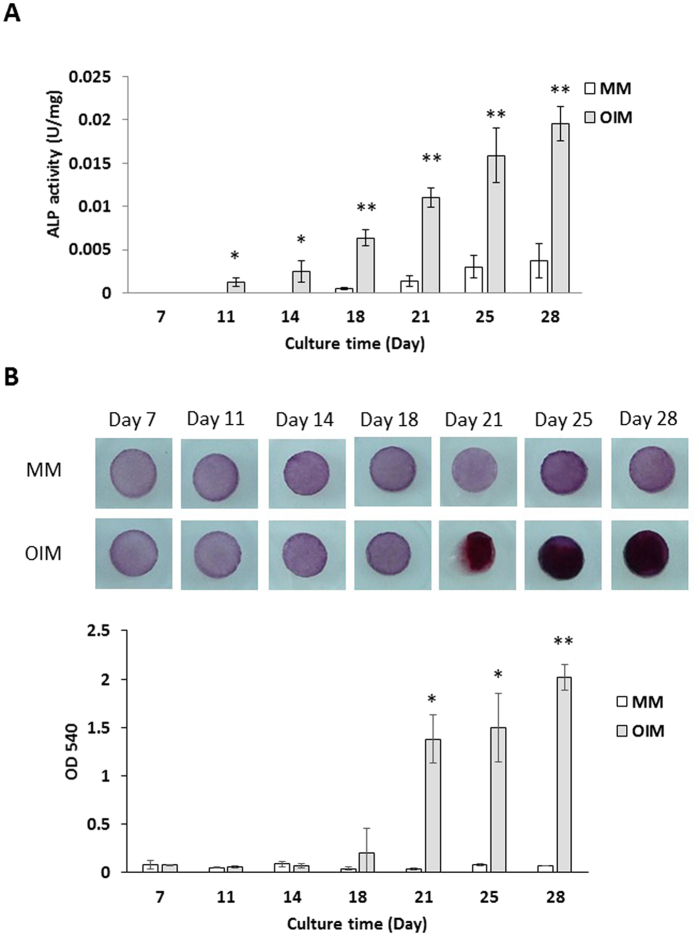
Osteogenic differentiation of preosteoblasts on the hemostatic gelatin sponge. (**A**) The ALP activity of preosteoblasts on the gelatin-based scaffold. The ALP activity of preosteoblasts was induced using OIM. (**B**) The calcium deposition was monitored by Alizarin red staining. The calcium deposition can be observed 21 days after induction using OIM. (n = 3; *P < 0.05; **P < 0.01 compared with MM at same time point; MM: maintained culture medium; OIM: osteogenic induction medium).

ALP is a general marker of osteogenic differentiation. Total protein from the preosteoblast-seeded hemostatic gelatin sponge was extracted at different time points to determine ALP activity. The ALP activity of preosteoblasts under osteogenic induction was detected after day 11, and increased with culture time (Fig. 8A). However, the ALP activity of preosteoblasts not receiving osteogenic induction was not detected until after day 18 and was much lower than the ALP activity of preosteoblasts under osteogenic induction.

We used Alizarin red staining to monitor calcium deposition and *in vitro* mineralization of preosteoblasts on the hemostatic gelatin sponge. As seen in Fig. 8B, the stained sponge with preosteoblasts cultured in OIM was dark red after 21 days, but no red color was observed for preosteoblasts cultured in MM. This was quantitatively supported by observations of optical density at 540 nm (OD540) for both culture conditions. This suggests that calcium deposition and *in vitro* mineralization of preosteoblasts on our hemostatic gelatin sponge was enhanced when they were cultured in OIM, and osteogenic differentiation was achieved.

## Discussion

This manuscript builds off our previous studies regarding cellular behaviors on different biomaterials^29^,^30^. Based on these studies, we employed a framework to examine the potential of a specific biomaterial, hemostatic gelatin sponge, for bone tissue engineering. Other biomaterials, e.g., collagen and its porous and denatured form, gelatin, have been widely used in tissue engineering studies because they are abundant, biodegradable, biocompatible, and have low immunogenicity^31^,^32^. Moreover, gelatin demonstrates several advantages including suitable solubility and less antigenicity when compared to its precursor^33^,^34^. In addition, signaling peptides, like the Arg–Gly–Asp (RGD) sequence of gelatin, can promote cell adhesion, migration, differentiation, and proliferation^34^. The tertiary structure of collagen, however, is denatured during hydrolysis, which decreases its structural variations, making it more convenient for further applications of gelatin^32^. It is clear that gelatin is useful as a scaffold for bone tissue engineering, but osteogenic differentiation studies employing it remained rare. Sisson *et al*. did explore such properties by employing an electrospinning process to fabricate gelatin scaffold with different fiber diameters and observed that MG63 cells differentiated faster on small fiber diameter scaffolds^18^. Gelatin is also advantageous because it can be easily integrated with a variety of materials and signaling molecules to improve their mechanical properties and functions^35^. For example, gelatin/CaSO_4_ scaffolds can support osteogenic differentiation of mesenchymal stem cells (MSCs) and can be used for bone regeneration^36^, and gelatin scaffolds incorporated with non-collagenous proteins have been shown to enhance osteogenic differentiation of MC3T3-E1 cells and improve bone regeneration^37^. Although many kinds of gelatin-based scaffolds have been developed for bone tissue engineering, the additive solvents and chemical modifications raise safety issues when entering clinical applications. These concerns usually limit the transfer of biomaterials from laboratory studies to clinical application. In order to solve this problem, and hasten the translational lag between research and clinical application, we chose to investigate the use of an already approved and clinically used gelatin material as a scaffold for bone tissue engineering.

In this study, we broke new ground by examining the use of a common hemostatic gelatin sponge as a scaffold for bone tissue engineering. In our degradation study, this hemostatic gelatin sponge maintained its structure when immersed in PBS for 8 weeks and a minor decrease of pH of PBS was observed. We assume this change is not important in an environment with larger buffer system, e.g., an *in vivo* physical environment. In the compression test, the compressive Young’s modulus of the hemostatic gelatin sponge was 170.81 ± 5.87 kPa, which is similar to a gelatin scaffold with similar pore size (pore size = 100–200 μm; compressive Young’s modulus = ~150 kPa)^38^. The compression strength of the gelatin sponge decreased after immersion in PBS for 8 weeks; however, the structure of the gelatin sponge remained intact. Moreover, while cultured with cells, the deposition of extracellular matrix on scaffold for tissue engineering may increase the compression strength, which may compensate for the loss of mechanical strength^38^. While stable in PBS, our hemostatic gelatin sponge demonstrated biodegradability through proteolytic reaction via pepsin and collagenase. Although these results may raise concerns about rapid gelatin sponge degradation, we should keep in mind that an *in vitro* assay with high concentrations of pepsin and collagenase was used to demonstrate the biodegradation of the gelatin sponge. Although this *in vitro* biodegradation assay is far from real physiological conditions, it may provide a quick and alternative method to understand gelatin sponge biodegradation in a short observation period. In addition, pepsin mainly exists in the stomach and is only used as a model enzyme to demonstrate biodegradation in this manuscript. Collagenase plays an important role in destroying extracellular structures. Generally speaking, the concentration of collagenase is quite low under normal conditions and only increases in an inflammatory status. For example, the collagenase concentration in the pulps of healthy people is 0.01 ± 0.009 ng/mL, however, collagenase concentration in the pulps of the patients with chronic pulpitis is 35 times higher than that of healthy people^39^. Similar overexpression of collagenase in inflammatory processes has also been reported in lumbar facet joint cartilage and synovial tissues of patients with lumbar spinal canal stenosis^40^. Moreover, the gelatin sponge used here induced no inflammatory reaction in the process of its degradation^20^. We can, then, assume that gelatin sponge biodegradation of the gelatin sponge will be slow in a real physiological environment. In addition, the hemostatic gelatin sponge demonstrated suitable biocompatibility to preosteoblasts. All of these findings suggested that hemostatic gelatin sponge was a promising scaffold for cell proliferation and osteogenic differentiation.

When culturing preosteoblasts on the hemostatic gelatin sponge, preosteoblasts attached well as confirmed by MTT assay, confocal microscopy, and SEM. It is worth mentioning that attached preosteoblasts showed spread-out morphology, cellular extensions, and a network between cells as confirmed by SEM. According to previous literature, the outgrown preosteoblast morphologies reflected typical fibroblast-like morphology, linking them to early-phase osteogenic marker expression in both mRNA and protein levels^41^. In addition, the more spread-out morphology of preosteoblasts under in a non-induced state than that under osteogenic induced state is similar to previous study, which may be contributed to different expression of osteoblastic markers and extracellular matrix production^42^. These results indicate that hemostatic gelatin sponge material provides an ideal environment for preosteoblast attachment.

When we studied osteogenic differentiation of preosteoblasts grown on the hemostatic gelatin scaffold, we observed that the growth of preosteoblasts plateaued on day 21. This plateau phenomenon may be attributable to limited cell growth space within the sponge. It is important to note that the migration of bone cell precursors to the site of skeletal injury plays an important role in bone repairing^43^. In our study, we observed higher migration ability for preosteoblasts under osteogenic induction as confirmed by examining sponge cross-sections under confocal microscopy. This phenomenon may be attributable to the fact that the migration of undifferentiated cells can be transiently upregulated early in osteogenic differentiation^44^. According to these observations, the hemostatic gelatin sponge provides an ideal environment for proliferation and migration of preosteoblasts.

ALP activity and calcium deposition were used to monitor osteogenic differentiation of preosteoblasts on the hemostatic gelatin sponge. The precise function of ALP is yet unknown, but it obviously plays an important role in calcium deposition and mineralization^45^,^46^. In our experiment, ALP activity was induced following 11 days of culture in OIM. The ALP induction of preosteoblasts on the hemostatic gelatin sponge is similar to tissue-culture-treated polystyrene dishe^47^. Using Alizarin red staining *in vitro* mineralization of preosteoblasts was observed on the hemostatic gelatin sponge after 21 days of culture in OIM. Similarly, a trend for calcium deposition in preosteoblasts grown on hemostatic gelatin sponge material can also be observed in preosteoblasts grown on tissue-culture-treated polystyrene dishes^47^. These results suggest that hemostatic gelatin sponge material is suitable for osteogenic differentiation, making it potentially useful for bone tissue engineering.

Scaffold pore size can influence tissue regeneration due to differences in cellular penetration and extracellular matrix between different cell types^48^. Scaffold pore size should be more than 100 μm in diameter to encourage the diffusion of essential nutrients and oxygen for cell survival^49^. For bone tissue engineering, the optimum pore size recommended for osteoconduction is between 100–400 μm^50^. The pore size of the hemostatic gelatin sponge used here was 148 ± 62 μm and the pore size reported in previous literature was 100–300 μm^21^, indicating that the pore size of this hemostatic gelatin sponge is suitable for osteoconduction.

Hemostatic gelatin sponge demonstrated many advantages for use in tissue engineering. It is easy to obtain, inexpensive, biodegradable, biocompatible, and is not known to induce allergic reactions or other undesirable side effects^24^. The gelatin sponge used here induced no inflammatory reaction in the process of its degradation when used as a scaffold for chondrocyte growth and cartilage tissue engineering in a rabbit model^20^. Further, gelatin sponge speckled with bone morphogenetic protein caused minimal tissue response when implanted into rat femoral muscle pouches^51^. Although gelatin sponge material has been demonstrated as a suitable *in vitro* model for the culture of chondrocytes, including human articular chondrocytes and bovine chondrocytes^21^,^22^,^23^, the reports of using this gelatin sponge as a scaffold for bone tissue engineering are still limited. Cegielski *et al*. attempted to use antlerogenic stem cells from deer antlers as an alternative to MSCs and demonstrated that gelatin sponge seeded with antlerogenic cells could be used to repair mandibular bone lesions in rabbits^25^. However, the fate and related osteogenic differentiation of antlerogenic stem cells within this gelatin sponge were not addressed. Some studies have tried to use gelatin sponges as a carrier or an implant for bone regeneration. For example, Paganelli *et al*. suggested using gelatin sponge as a carrier of stem cells in the alveoloplasty of cleft palate^24^. Arias-Gallo *et al*. used this gelatin sponge as a carrier of fibroblast growth factors for bone regeneration^26^. In addition, Skogh *et al*. applied gelatin sponge as an implant for repairing bone defect^27^. However, the data were limited and the potential for osteogenic differentiation of cells on gelatin sponge was not addressed. Our study shows, for the first time, that osteogenic differentiation can be achieved on gelatin sponge material and indicates the potential of gelatin sponge material for bone tissue engineering.

As a mechanically durable 3D cell culture system, cellulose papers have recently become a popular material for study^52^,^53^,^54^. Cellulose papers have been successfully applied to create 3D structures that allow for the examination of cell response to oxygen and nutrient gradients in a high-throughput manner by stacking layers of paper with cells; subsequent de-stacking has provided further valuable information. Although cellulose papers are inexpensive, easy to obtain, and eco-friendly, the attachment of cells to cellulose papers in these studies is usually achieved through using extracellular matrix, e.g. Matrigel. In our experience, preosteoblasts can attach to cellulose papers but show rounded morphology without Matrigel. In addition, the proliferation of preosteoblasts on cellulose paper is very slow, indicating that cellulose papers may not be suitable for cell culture without Matrigel (our unpublished data). This would limit the use of cellulose papers in cell culture.

Hemostatic gelatin sponges may provide a novel 3D structure for cells to proliferate, migrate, and differentiate under nutrient and growth factor gradients. For example, our results demonstrated migration of preosteoblasts to the center of our gelatin sponge at an early cell culture stage. Following long-term culture, more preosteoblasts were observed near the surface of the hemostatic gelatin sponge and less preosteoblasts were found within the hemostatic gelatin sponge as confirmed by examinations of hemostatic gelatin sponge cross-sections via confocal microscopy. This dynamic homeostasis of cell distribution can be attributed to nutrient and bio-signal gradients between surface and center of the hemostatic gelatin sponge. This condition mimics real physiological environment more closely and prompts greater investigation. According to our experimental observations, hemostatic gelatin sponge material demonstrated many advantages including its potential as a 3D system for cell culture.

In the future, hemostatic gelatin sponge material could be further used to guide bone regeneration. A preosteoblast- preloaded sponge could act as a barrier to prevent soft-tissue invasion into the bone defect and form a chamber to guide and enhance the process of bone regeneration^55^. While we have achieved highly encouraging results, we hope to improve the mechanical properties of the hemostatic gelatin sponge used by combining it with bioceramics to further the fabrication of tissue-engineered bone.

Our study described, for the first time, the potential of a hemostatic gelatin sponge, Spongostan, to act as a scaffold for bone tissue engineering. We demonstrated its suitability for biodegradability, biocompatibility, cellular proliferation, cellular migration, and osteogenic differentiation of preosteoblasts. In addition, this sponge may provide a novel 3D cell culture system to monitor dynamic homeostasis of cell distribution, a process that more closely mimics the real physiological environment. We believe that this study and these results offer a valuable seed of information that may be used to promote the use of hemostatic gelatin sponges and other common clinical biomaterials for versatile clinical uses.

## Methods

### Materials

The hemostatic gelatin sponge, Spongostan (Ferrosan Medical Device, MS0003), was chosen as a scaffold for this study. We cut the hemostatic gelatin sponge into a disk shape with a diameter of 8 mm and a thickness of 1 mm for the following studies. All reagents for cell culture were purchased from Life Technologies (Grand Island, NY, USA).

### Degradation assay

We immersed the hemostatic gelatin sponge disks in 1 mL PBS (Corning, 21-040-CV) and incubated them at 37 °C for 8 weeks. We removed the disks and collected the immersion solution for pH analysis. We washed the disks three times with ddH_2_O. The disks then were vacuum-dried and their remaining dry weight was measured.

To determine biodegradation characteristics, we immersed the disks in 1.5 mL pepsin solution containing 10 ng/mL pepsin (Sigma, P7000) in 0.01N HCl or collagenase solution consisting of 1 μg/mL collagenase (Sigma, C5138) in PBS and then incubated them at 37 °C. The disks immersed in 0.01N HCl or PBS (without pepsin or collagenase) were used as control for biodegradation. In this study, we treated the disks as indicated without refreshing solutions. At a predetermined time, we collected and washed them three times with ddH_2_O. The disks were then vacuum-dried and their resulting weight was measured.

### Compression test

To determine mechanical characteristics, we immersed the hemostatic gelatin sponge disks in 1 mL PBS and incubated them at 37 °C for 8 weeks without refreshing solutions. At a predetermined time, we collected the disks and performed compression tests using an Ultra Small-capacity Load Cell (KYOWA, LTS-200GA) and a stage controller (SHOT-204MS). The compressive rate was 5 μm/sec for all measurements. The disks were compressed to ∼60% of their original thickness. We calculated Young’s modulus of each disk to monitor the mechanical changes of the disks immersed in PBS for 8 weeks.

### Cell culture

We used MC3T3-E1 cell line preosteoblasts derived from *Mus musculus* to study *in vitro* osteoblast differentiation^56^. MC3T3-E1 cells were cultured in maintained culture medium (MM), including minimum essential medium α supplemented with 10% heat-inactivated fetal bovine serum, 100 U/mL penicillin, 100 μg/mL streptomycin, 50 μg/mL gentamycin, and 250 ng/mL fungizone. Cell cultures were maintained in a humidified incubator at 37 °C and 5% CO_2_.

### Biocompatibility assay

The biocompatibility assay protocol was modified according to international standard ISO10993-5. Briefly, we immersed six gelatin sponge disks in 1 mL of MC3T3-E1 MM for 24 hours at 37 °C to obtain a sample extract. We seeded MC3T3-E1 cells into wells of a 96 well plate at a concentration of 10^4^ cells per well and incubated them overnight at 37 °C. we then added a set of sample extract serial dilutions into each of the cell-seeded wells. All experiments were performed in triplicate. After culturing for 24 hours at 37 °C, cytotoxicity was determined using MTT assay. MC3T3-E1 MM without exposed gelatin sponge disk was use as internal control for this study.

### Osteogenic differentiation of preosteoblasts

MC3T3-E1 cells were seeded onto each hemostatic gelatin sponge disks at a concentration of 5 × 10^3^ cells per disk. To induce osteogenic differentiation of MC3T3-E1 cells, osteogenic induction medium (OIM), MC3T3-E1 MM with 10^−7 ^M dexamethasone (Sigma, D8893), 10 mM β-Glycerol phosphate (Sigma, G9422), and 50 μg/mL ascorbic acid (Sigma, A4544), was applied one day after seeding of MC3T3-E1 cells and medium was changed with fresh medium every 2~3 days^57^. In addition, we used MC3T3-E1 MM for comparison in this study.

### Confocal microscopy

To examine cell attachment, we collected and washed the hemostatic gelatin sponge disks with PBS one day after cell seeding. To examine osteogenic induction, we seeded 5 × 10^3^ MC3T3-E1 cells onto each gelatin disk, and then collected the disks at different time points (day 7, 11, 14, 18, 21, 25, and 28) and washed with PBS. We labeled live and dead cells with fluorescent LIVE/DEAD viability/cytotoxicity probes (Thermo Fisher Scientific, Molecular Probes, L3224), and cell proliferation was monitored using a confocal microscope (Leica, TCS SP2).

### Scanning electron microscopy

To examine cell morphology, the hemostatic gelatin sponge disks were washed three times with ice-cold PBS and fixed in a mixed solution of 3% glutaraldehyde, and 2% paraformaldehyde in 0.1 M cacodylate buffer at 4 °C for 2 hours, followed by 0.5% glutaraldehyde fixing at 4 °C overnight. The disks were vacuum-dried overnight, coated with platinum–palladium sputtering, and examined with a scanning electronic microscope (Hitachi, S-5000). We used ImageJ (1.49v) to measure pore diameter of the hemostatic gelatin sponge in SEM images.

### Alkaline phosphatase assay

MC3T3-E1 cells were seeded onto each hemostatic gelatin sponge disks at a concentration of 5 × 10^3^ cells per disk and then cultured using OIM as previously described. The disks with MC3T3-E1 cells were collected and washed twice with PBS at different time points. The total protein from each disk seeded with MC3T3-E1 cells was extracted using RIPA buffer (Sigma, R0278) containing phosphatase inhibitor cocktail 2 (Sigma, P5726), phosphatase inhibitor cocktail 3 (Sigma, P0044) and protease inhibitor cocktail (Sigma, P8340). The alkaline phosphatase activity was determined via colorimetry using an alkaline phosphatase assay kit (Abcam, ab83369).

### Calcium deposition assay

The hemostatic gelatin sponge disks used to examine the osteogenic differentiation of preosteoblasts were washed twice with PBS before being fixed with 4% paraformaldehyde in PBS at room temperature for 15 minutes. Subsequently, we removed the solution and washed the disks three times with ddH_2_O. Two percent Alizarin red S staining solution (Sciencell, 0223) was applied and disks were incubated at room temperature for 30 minutes. The staining agent was removed and the disks were washed 3–5 times with ddH_2_O. The disks were photographed using a digital camera (Panasonic, DMC-FX36GT). To quantify the result of the Alizarin red S staining, the dye was desorbed using 10 wt% cetylpyridinium chloride (Sigma, C9002) for 1 hour and absorbance was read at 540 nm using an ELISA reader (Molecular Devices, SpectraMax M5)^58^.

### Statistical analysis

All experimental data are expressed as the mean ± SD, unless otherwise stated. The significance of differences between groups was analyzed using the Student’s *t* test. P < 0.05 was considered statistically significant.

## Additional Information

**How to cite this article**: Kuo, Z.-K. *et al*. Osteogenic differentiation of preosteoblasts on a hemostatic gelatin sponge. *Sci. Rep.* **6**, 32884; doi: 10.1038/srep32884 (2016).

## Supplementary Material

Supplementary Information
