# Supplementary material for: Osteogenic differentiation of preosteoblasts on a hemostatic gelatin sponge
Source: Sci Rep. 2016 Sep 12;6:32884. doi: 10.1038/srep32884 (PMC5018723; doi:10.1038/srep32884)
Supplement: Supplementary Information [file srep32884-s1.doc]

# Osteogenic differentiation of preosteoblasts on a hemostatic gelatin sponge

**Zong-Keng Kuo^1,2,†^, Po-Liang Lai^3,†^, Elsie Khai-Woon Toh^3^, Cheng-Hsi Weng^4^, Hsiang-Wen Tseng^2^, Pei-Zen Chang^4^, Chih-Chen Chen^1^ and Chao-Min Cheng^5, *^**

^1^Institute of Nanoengineering and Microsystems, National Tsing Hua University, Hsinchu City 30013, Taiwan

^2^Pharmacodynamics Technology Department, Center of Excellence for Drug Development, Biomedical Technology and Device Research Labs, Industrial Technology Research Institute, Hsinchu City 30011, Taiwan

^3^Department of Orthopedic Surgery, Bone and Joint Research Center, Chang Gung Memorial Hospital, College of Medicine, Chang Gung University, Taoyuan 33305, Taiwan

^4^Institute of Applied Mechanics, National Taiwan University, Taipei City 10617, Taiwan

^5^Institute of Biomedical Engineering, National Tsing Hua University, Hsinchu City 30013, Taiwan

**^†^**These authors contributed equally

***Corresponding Author**

Dr. Chao-Min Cheng

Institute of Biomedical Engineering, National Tsing Hua University, Hsinchu City 30013, Taiwan

Phone: +886-35162402

Fax: +886-35745454

E-mail address: chaomin@mx.nthu.edu.tw

**Supplementary Information**

**Degradation assay in ddH_2_O**

In addition to examine biodegradation of hemostatic gelatin sponge in PBS, we also tested the biodegradation of sponge disks in ddH_2_O. Sponge disks were immersed in ddH_2_O and maintained in an incubator at 37°C for 8 weeks. We collected the immersion solution for pH analysis every two weeks. Additionally, we washed the disks three times with ddH_2_O. The disks then were vacuum-dried and their resulting dry weight was measured. Hemostatic gelatin sponge condition was observed each week (Supplemental Figure 1A). No structural decay was observed, suggesting sponge structure stability under these conditions. The remaining weight of the sponge decreased over time (Supplemental Figure 1B), and the sponge weight at week 8, which indicated that they had maintained 83.7±2.9% of their original weight, was similar to the weight maintenance results following immersion in PBS (84.9 ± 3.9 %). The pH changed during this time span from 4.41 ± 0.02 to 4.63 ± 0.01 (Supplemental Figure 1C). The pH of ddH_2_O without a sponge was 5.13± 0.01 at the end of study. Similar to the phenomenon using PBS, a change of pH may be attributable to dissolution of CO_2_ in ddH_2_O. However, a higher acidification of ddH_2_O exposed to a sponge was observed when compared to using PBS, which is likely attributable to the higher buffer ability of PBS.

**
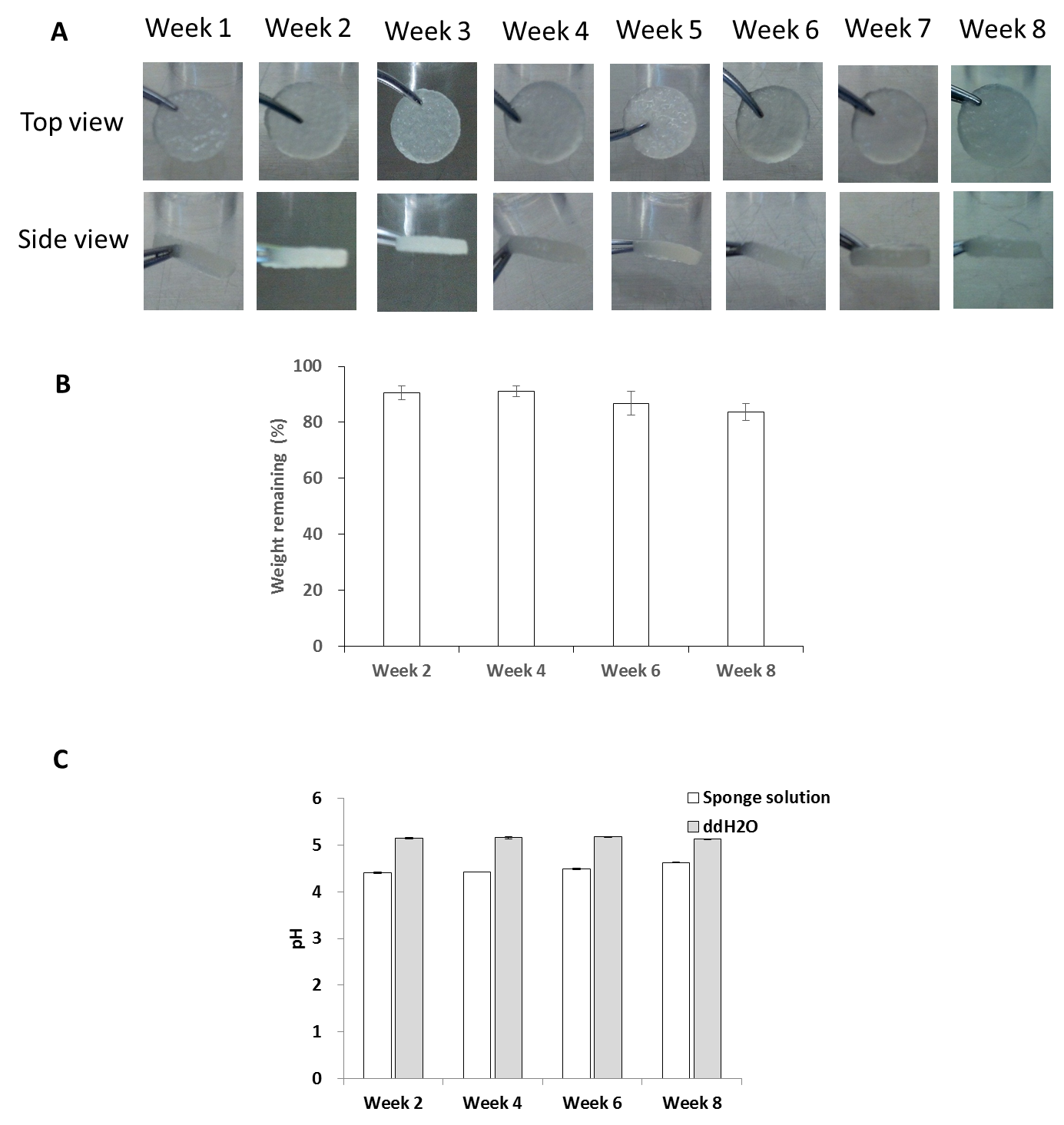
**

**Supplemental Figure 1.** The degradation of the hemostatic gelatin sponge when immersed in ddH_2_O. (A) Photographs of the sponge at different time points. The hemostatic gelatin sponge maintained its structure for 8 weeks. (B) The resulting weight of the sponge after immersion in ddH2O. The resulting weight at week 8 shows that the sponge maintained 83.7±2.9% of its original weight. (C) The pH of the solution around the sponge.The pH is 4.63 ± 0.01 at the 8th week. n=6.
